# Supplementary material for: Association between Daytime Sleepiness, Fatigue and Autonomic Responses during Head-Up Tilt Test in Multiple Sclerosis Patients
Source: Brain Sci. 2023 Sep 19;13(9):1342. doi: 10.3390/brainsci13091342 (PMC10526123; doi:10.3390/brainsci13091342)
Supplement: Supplementary file 1 [file brainsci-13-01342-s001.zip › brainsci-2598320-supplementary.pdf]

Table S1. Relationships between Daily Sleepiness, Fatigue Symptoms and cardiac autonomic parameters.

Variable	CFQ Score		ESS Score	
	R	*p*-Value	R	*p*-Value
CFQ [score]	−0.03	0.826	0.17	0.212
EDSS	0.27	**0.04**	−0.11	0.402
Disease duration [years]	0.09	0.517	−0.11	0.416
Age [years]	0.01	0.947	−0.33	**0.011**
ESS [score]	0.17	0.212		
HR [1/min]	−0.2	0.137	−0.18	0.165
sBP [mmHg]	0.14	0.288	−0.1	0.46
dBP [mmHg]	0.05	0.698	−0.15	0.264
mBP, [mmHg]	0.09	0.515	−0.14	0.296
CI [L/(min·m^2)]	−0.07	0.58	0.18	0.182
TPRI [dyn·s·m^2/cm^5]	0.1	0.439	−0.2	0.141
LFnu-RRI [%]	−0.21	0.105	−0.17	0.215
HFnu-RRI [%]	0.22	0.099	0.16	0.217
PSD-RRI [ms^2]	0.09	0.496	0.21	0.113
LF/HF-RRI [1]	−0.23	0.088	−0.18	0.175
LF/HF [1]	−0.17	0.195	−0.28	**0.033**
LFnu-dBP [%]	−0.05	0.705	−0.32	**0.013**
HFnu-dBP [%]	0.13	0.324	0.23	0.08
PSD-dBP [mmHg^2]	−0.16	0.244	−0.04	0.786
LFnu-sBP [%]	−0.2	0.127	−0.42	**0.001**
HFnu-sBP [%]	0.04	0.738	0.17	0.201
PSD-sBP [mmHg^2]	−0.02	0.886	−0.24	0.073
BRS [ms/mmHg]	−0.03	0.826	0.23	0.093
ΔPHASE$_1$ HR [1/min]	0.14	0.307	0.14	0.297
ΔPHASE$_1$ sBP [mmHg]	−0.03	0.813	0.07	0.623
ΔPHASE$_1$ dBP [mmHg]	−0.05	0.711	0.01	0.961
ΔPHASE$_1$ mBP [mmHg]	−0.05	0.711	0.03	0.828
ΔPHASE$_1$ CI [L/(min·m^2)]	0.05	0.706	−0.12	0.371
ΔPHASE$_1$ TPRI [dyn·s·m^2/cm^5]	0.1	0.463	0.04	0.761
ΔPHASE$_1$ LFnu-RRI [%]	0.07	0.599	0.17	0.204
ΔPHASE$_1$ HFnu-RRI [%]	−0.09	0.515	−0.18	0.172
ΔPHASE$_1$ PSD-RRI [ms^2]	0.09	0.479	−0.17	0.196
ΔPHASE$_1$ LF/HF-RRI [1]	0.02	0.857	0.18	0.169
ΔPHASE$_1$ LF/HF [1]	0.03	0.819	0.17	0.192
ΔPHASE$_1$ LFnu-dBP [%]	−0.15	0.25	0.1	0.461
ΔPHASE$_1$ HFnu-dBP [%]	−0.04	0.75	−0.21	0.119
ΔPHASE$_1$ PSD-dBP [mmHg^2]	0.22	0.092	0	0.975
ΔPHASE$_1$ LFnu-sBP [%]	0.15	0.266	0.1	0.445
ΔPHASE$_1$ HFnu-sBP [%]	0.11	0.415	−0.08	0.54
ΔPHASE$_1$ PSD-sBP [mmHg2]	−0.08	0.552	0.19	0.156
ΔPHASE$_1$ BRS [ms/mmHg]	0.08	0.548	−0.21	0.114
ΔPHASE$_2$ HR [1/min]	0.09	0.516	0.14	0.286
ΔPHASE$_2$ sBP [mmHg]	0.15	0.275	0.16	0.24
ΔPHASE$_2$ dBP [mmHg]	0.15	0.251	0.18	0.169
ΔPHASE$_2$ mBP [mmHg]	0.13	0.321	0.2	0.127
ΔPHASE$_2$ CI [L/(min·m^2)]	0.04	0.746	−0.1	0.466
ΔPHASE$_2$ TPRI [dyn·s·m^2/cm^5]	0.15	0.269	0.13	0.349

ΔPHASE$_2$ LFnu-RRI [%]	−0.02	0.863	0.15	0.269
ΔPHASE$_2$ HFnu-RRI [%]	0.02	0.902	−0.15	0.246
ΔPHASE$_2$ PSD-RRI [ms^2]	0.02	0.853	−0.26	**0.047**
ΔPHASE$_2$ LF/HF-RRI [1]	−0.31	**0.016**	−0.07	0.596
ΔPHASE$_2$ LF/HF [1]	−0.36	**0.005**	−0.07	0.613
ΔPHASE$_2$ LFnu-dBP [%]	−0.09	0.52	0.2	0.125
ΔPHASE$_2$ HFnu-dBP [%]	0.12	0.365	−0.22	0.099
ΔPHASE$_2$ PSD-dBP [mmHg^2]	0.22	0.097	−0.06	0.678
ΔPHASE$_2$ LFnu-sBP [%]	0.17	0.199	0.12	0.375
ΔPHASE$_2$ HFnu-sBP [%]	0.16	0.239	−0.07	0.614
ΔPHASE$_2$ PSD-sBP [mmHg^2]	−0.06	0.657	0.09	0.494
ΔPHASE$_2$ BRS [ms/mmHg]	0.08	0.524	−0.23	0.079
ΔPHASE$_3$ HR [1/min]	0.02	0.898	0.12	0.361
ΔPHASE$_3$ sBP [mmHg]	0.13	0.314	0.13	0.317
ΔPHASE$_3$ dBP [mmHg]	0.09	0.505	0.28	**0.034**
ΔPHASE$_3$ mBP [mmHg]	0.09	0.477	0.24	0.073
ΔPHASE$_3$ CI [L/(min·m^2)]	0.02	0.854	−0.11	0.419
ΔPHASE$_3$ TPRI [dyn·s·m^2/cm^5]	0.12	0.386	0.12	0.354
ΔPHASE$_3$ LFnu-RRI [%]	0.06	0.635	0.22	0.093
ΔPHASE$_3$ HFnu-RRI [%]	−0.06	0.635	−0.22	0.093
ΔPHASE$_3$ PSD-RRI [ms^2]	−0.12	0.372	−0.15	0.257
ΔPHASE$_3$ LF/HF-RRI [1]	−0.16	0.226	0.1	0.456
ΔPHASE$_3$ LF/HF [1]	−0.21	0.117	0.08	0.554
ΔPHASE$_3$ LFnu-dBP [%]	−0.14	0.31	0.13	0.341
ΔPHASE$_3$ HFnu-dBP [%]	−0.04	0.782	−0.15	0.261
ΔPHASE$_3$ PSD-dBP [mmHg^2]	0.15	0.252	0.04	0.771
ΔPHASE$_3$ LFnu-sBP [%]	0.12	0.363	0.05	0.69
ΔPHASE$_3$ HFnu-sBP [%]	0.04	0.764	−0.03	0.819
ΔPHASE$_3$ PSD-sBP [mmHg^2]	0.01	0.938	0.07	0.605
ΔPHASE$_3$ BRS [ms/mmHg]	0.04	0.772	−0.21	0.112
ΔPHASE$_4$ HR [1/min]	0.06	0.644	0.11	0.417
ΔPHASE$_4$ sBP [mmHg]	0.18	0.176	0.09	0.505
ΔPHASE$_4$ dBP [mmHg]	0.21	0.109	0.28	**0.034**
ΔPHASE$_4$ mBP [mmHg]	0.2	0.137	0.22	0.09
ΔPHASE$_4$ CI [L/(min·m^2)]	0.04	0.781	−0.07	0.606
ΔPHASE$_4$ TPRI [dyn·s·m^2/cm^5]	0.11	0.402	0.09	0.489
ΔPHASE$_4$ LFnu-RRI [%]	0.04	0.762	0.24	0.07
ΔPHASE$_4$ HFnu-RRI [%]	−0.04	0.769	−0.24	0.069
ΔPHASE$_4$ PSD-RRI [ms^2]	−0.09	0.521	−0.23	0.081
ΔPHASE$_4$ LF/HF-RRI [1]	−0.17	0.214	0.11	0.416
ΔPHASE$_4$ LF/HF [1]	−0.26	0.045	0.05	0.736
ΔPHASE$_4$ LFnu-dBP [%]	−0.1	0.46	0.09	0.524
ΔPHASE$_4$ HFnu-dBP [%]	0.01	0.961	−0.19	0.157
ΔPHASE$_4$ PSD-dBP [mmHg2]	0.16	0.231	−0.06	0.63
ΔPHASE$_4$ LFnu-sBP [%]	0.14	0.287	0.11	0.429
ΔPHASE$_4$ HFnu-sBP[%]	0.03	0.818	−0.01	0.953
ΔPHASE$_4$ PSD-sBP [mmHg2]	0.03	0.84	0.04	0.76
ΔPHASE$_4$ BRS [ms/mmHg]	0.13	0.337	−0.21	0.116

HR—heart rate; sBP—systolic blood pressure; dBP—diastolic blood pressure; mBP—mean blood pressure; CI—cardiac index; TPRI—total peripheral index; LFnu-RRI—low frequency R-R interval; in normalized units; HFnu-RRI—high-frequency R-R interval in normalized units; PSD-RRI—power spectral density R-R interval; LF/HF—ratio between low and high band for heart rate and blood pressure variability; PSD-sBP—power spectral density of systolic blood pressure variability;

LFnu-sBP—low frequency of systolic blood pressure variability in normalized units; HFnu-sBP—high frequency of systolic blood pressure variability in normalized units; PSD-dBP—power spectral density of diastolic blood pressure variability; LFnu-dBP – low frequency of diastolic blood pressure variability in normalized units; HFnu-dBP—high frequency of diastolic blood pressure variability in normalized units; BRS—baroreflex sensitivity; ESS score – excessive daily sleepiness; CFQ score-fatigue severity; ΔPHASE-baseline.
